# A review on methods for diagnosis of breast cancer cells and tissues

**DOI:** 10.1111/cpr.12822

**Published:** 2020-06-12

**Authors:** Ziyu He, Zhu Chen, Miduo Tan, Sauli Elingarami, Yuan Liu, Taotao Li, Yan Deng, Nongyue He, Song Li, Juan Fu, Wen Li

**Affiliations:** ^1^ Hunan Key Laboratory of Biomedical Nanomaterials and Devices Hunan University of Technology Zhuzhou China; ^2^ State Key Laboratory of Bioelectronics School of Biological and Medical Engineering Southeast University Nanjing China; ^3^ Surgery Department of Galactophore Central Hospital of Zhuzhou City Zhuzhou China; ^4^ School of Life Sciences and Bioengineering (LiSBE) The Nelson Mandela African Institution of Science and Technology (NM‐AIST) Arusha Tanzania; ^5^ Hunan Provincial Key Lab of Dark Tea and Jin‐hua School of Materials and Chemical Engineering Hunan City University Yiyang China; ^6^ School of Medicine South China University of Technology Guangzhou China

## Abstract

Breast cancer has seriously been threatening physical and mental health of women in the world, and its morbidity and mortality also show clearly upward trend in China over time. Through inquiry, we find that survival rate of patients with early‐stage breast cancer is significantly higher than those with middle‐ and late‐stage breast cancer, hence, it is essential to conduct research to quickly diagnose breast cancer. Until now, many methods for diagnosing breast cancer have been developed, mainly based on imaging and molecular biotechnology examination. These methods have great contributions in screening and confirmation of breast cancer. In this review article, we introduce and elaborate the advances of these methods, and then conclude some gold standard diagnostic methods for certain breast cancer patients. We lastly discuss how to choose the most suitable diagnostic methods for breast cancer patients. In general, this article not only summarizes application and development of these diagnostic methods, but also provides the guidance for researchers who work on diagnosis of breast cancer.

## 1 INTRODUCTION

Breast cancer (BC) has become one of the most common malignant tumours, and latest dates from CA‐cancer magazine show that the incidence rate is increasing every year. In 2019, approximately 316 700 new cases of BC have been confirmed in US women, and the growth rate is nearly 0.3% per year.^1^ The data from China show that the incidence rate of BC also raises per year (272 400 cases in 2015 and 367 900 cases in 2018).^2^, ^3^ Taking population growth into consideration, experts predict that there will be about 3.2 million new BC cases per year globally by 2050.^4^ More notably, not only the number of patients with BC is increasing all over the world, but also the age of affected patients is tending to be younger.^5^ There are many factors causing above situation, such as age, family history, lifestyle environments and so on.^4^, ^6^, ^7^ The high incidence rate of BC is unavoidable, but decreasing the mortality of BC is feasible. Early detection and treatment are critical to curing BC, because it tends to metastasize in the middle and last stage.^8^, ^9^, ^10^ Therefore, finding BC is vital in early stage, which can greatly improve the survival rate of patients.

To quickly and accurately screen BC, many diagnostic methods based on imaging and molecular biotechnology have been developed. It is indispensable to summarize and evaluate these methods, to provide value information for clinical diagnosis. Jafari^11^ summarized various imaging techniques and biochemical biomarkers used for detection and monitoring BC patients and highlighted that it is helpful to diagnose and treat patients with BC by measuring level of certain biomarkers. Weaver^12^ described definitions and applications of imaging “biomarkers,” and thought that they can build the decision support system by these markers to provide help for clinical breast care and BC–related research. Many articles review these methods for diagnosing BC mainly from these aspects, by introducing the contribution of imaging techniques (including molecular imaging markers) in diagnosing BC patients, and summarizing these findings on connection between newly discovered tumour makers and BC patients.^13^, ^14^, ^15^ Many articles describe a large number of diagnostic methods for breast cancer, but few articles introduce how to choose suitable diagnostic methods for different types of BC patients.

In this review, many diagnostic methods are reviewed, such as mammography (MG), ultrasonography (US), magnetic resonance imaging (MRI), nucleic acid hybridization system (NAHS), real‐time fluorescence quantitative PCR system (RT‐qPCR), protein hybridization system (PHS), flow cytometer (FCM) and so on. We herein introduce their development and summarize their advantages and disadvantages and provide some diagnostic schemes for different types of BC patients. The article can help future research and development in diagnosing BC patients and guiding people who are working on BC research, on how to choose the suitable methods for diagnosing BC patients.

## 2 IMAGING DIAGNOSIS

Utilization of imaging techniques shows clearly the morphology and location of tumour tissues and proves much clinical information that is valuable to doctors. However, imaging techniques may cause harm to patients when using contrast agents and high energy rays. Therefore, we should discuss these imaging techniques and choose the most appropriate diagnostic method for BC patients. These imaging techniques mainly include mammography (MG), ultrasonography (US), magnetic resonance imaging (MRI), positron emission computed tomography (PET), computed tomography (CT) and single‐photon emission computed tomography (SPECT). In Table 1, we list the advantages and disadvantages of these imaging methods. In view of high cost, poor practicability and radiation damage, PET, CT and SPECT are not recommended in diagnosing BC patients. However, these techniques can be used as auxiliary diagnostic methods for diagnosing BC in some special cases, such as screening for metastatic BC, presence of bone and lymphatic metastases. Therefore, we only introduce MG, US and MRI that are preferred methods for screening BC. Summary and evaluation of these common imaging techniques will help doctors to better serve patients and promote the development of clinical diagnosis.

**Table 1:** Advantages and disadvantages of imaging techniques

Imaging techniques	Advantages	Disadvantages
XRM	The golden standard for diagnosing BC patients Suitable as a screening method for BC Finding mammary gland calcification	Not suitable for people under 40 Not suitable for people with high gland density No more than twice a year
US	Suitable screening for young women Non‐invasive diagnostic methods Finding mammary gland inflammation	Not suitable for small mass and atypical tissue Affected by the examining doctor Definition and Resolution are not high
MRI	High sensitivity and specificity to invasive BC Screening of high‐risk groups, such as family history of BC Suitable for patients with breast‐conserving surgery	Not for everyone, such as patients with Claustrophobia and hypersensitivity to contrast Not suitable for wide scale screening Not suitable for BC staging
PET	High sensitivity to BC recurrence and metastasis Helpful for staging of the BC High sensitivity to small breast tumour (>0.5 cm)	High cost, not recommended as routine screening Not suitable for patients with hypersensitivity to Developer
CT	Supplementary diagnostic method for BC, such as identifying BC with or without intrapulmonary metastases	Not the first choice for diagnosing BC Radiation damage Poor spatial resolution and need experienced doctor
SPECT	High resolution, small field of vision Recommended use when suspects metastasis (such as osseous metastasis)	Obtaining littler clinic information Not suitable for patients with inflammatory bone lesions and bone proliferative metabolic abnormalities or variations

Abbreviations: CT, Computed tomography; MRI, Magnetic resonance imaging; PET, Positron emission tomography; SPECT, Single‐photon emission computed tomography; US, Ultrasonography; XRM, X‐ray mammography.

### 2.1 Mammography

Mammography (MG) is preferred strategy for screening and diagnosing BC and helps doctors obtain clinic information on BC patients. The evidence suggests that the mortality rate of BC patients could be reduced 30%‐40% though early MG screening.^16^ Meanwhile, the diagnostic result of MG is only positive criteria for 4%‐10% of BC patients (eg, patients who exhibited only slight calcification).^17^, ^18^ MG is developed continuously with passage of time. Contrast‐enhanced mammography (CEM) and digital breast tomosynthesis (DBT) are at present two main strategies that diagnose BC patients in clinic.^19^, ^20^ Through investigation, CEM is superior to full‐field digital mammography (FFDM), and the value of CEM in diagnostic accuracy and evaluation of disease extent is close to breast MRI.^21^, ^22^ Similarly, DBT also has good performance, such as higher specificity, when compared with FFDM (96.4%, 57229/59381% vs 97.5%, 23427/24020, *P* < .001).^23^ In 1998, computer‐aided detection (CAD) was developed and it greatly improved the sensitivity of instruments from about 60% to 100%.^24^ CEM can be combined with CAD to diagnose BC patients, and it could carry out classification for breast masses, and the ROC curves for patients will be significantly increased to 0.848 ± 0.038 (*P* < .01).^25^ Similarly, the reading time for DBT can be improved to about 29.2%, and the ROC curves for patients will be increased from 0.841 to 0.850 (95% CI, −0.012 to 0.030) when combined with CAD.^26^

In general, MG and its derivatives are indispensable part in diagnosis and screening of BC patients. Their advantages are as follows: rapid screening, high accuracy, low cost and suitable for promoted use. Therefore, MG is optimal imaging diagnostic method for patients with low income and eliminates the risk for BC, etc However, these factors may cause MG to not be suitable for everyone. For example, MG needs harmful contrast agent and X‐ray to do imaging, so cannot be used repeatedly in a short period of time, and is not recommended to use for patients under age of 40.^27^ In the future, MG will tend to be harmless and with high resolution. Meanwhile, with advancement of artificial intelligence (AI) technique and development of sensors, it is viable to realize automation of detection and analysis of BC.

### 2.2 Ultrasonography

Ultrasonography (US) is applied in observing morphology and variation condition of tumour tissues, and it can accurately locate the location of lesions. US is not harmful to humans and is suitable for everyone. The development history of US is as follows: the early grayscale US only showed whether the tumour existed at detection site, but it was difficult to distinguish benign and malignant tumours, because its resolution was low.^28^, ^29^ Surely, the two‐dimensional US only gets some flat images of tumour, and judgement by physicians will be affected. So, three‐dimensional US technology was developed for three‐dimensional imaging of tumour morphology and blood vessel distribution, which are shown when patients are diagnosed.^30^ The colour Doppler US is one of many three‐dimensional US and can clearly reflect the situation of tumour and blood flow information and provide doctors with more valuable clinical information, so that it can distinguish benign and malignant tumours.^31^ In 1998, Krouskop^32^ found that there are elastic differences in different tissues, which provides theoretical foundation for developing elastic US. Moreover, some researches screened the suspected pathological tissues by using elastic US and found that it improves greatly the accuracy for diagnosing BC.^33^, ^34^ However, when combined with three‐dimensional US, the elastic US can diagnose axillary lymphadenopathy and classify the patient's tumour state.^35^ Though MG is optimal method to detect the calcification condition of BC, when the size of calcification is too small, it is difficult to be detected by MG or routine US.^36^ A new US image‐processing technique, MicroPure, was therefore developed. This method can reduce speckle by analysing pictures of spatial feature and frequency and create images that have high contrast resolution and high tissue uniformity.^37^ Machado et al^38^ processed ex vivo surgical breast specimens by using MicroPure examination and found that the MicroPure has high recognition rate to microcalcifications of BC, and conventional US cannot found its situation.

US has many advantages, such as use of few contrast agents, none high energy rays and suitability for all ages. Meanwhile, when MG cannot be used, US can become an alternative diagnostic method for BC. However, the US has limitations that need professional operation and lower definition and resolution than CT. Notably, the people who are obese and those with nodi lymphatici parasternales metastasis are not suitable to use US for diagnosis. In the future, intelligent US detection will be a new tendency, which will greatly reduce errors due to unprofessional judgements, thereby helping doctors to get more accurate diagnostic results.

### 2.3 Magnetic resonance imaging

**Figure 1:**
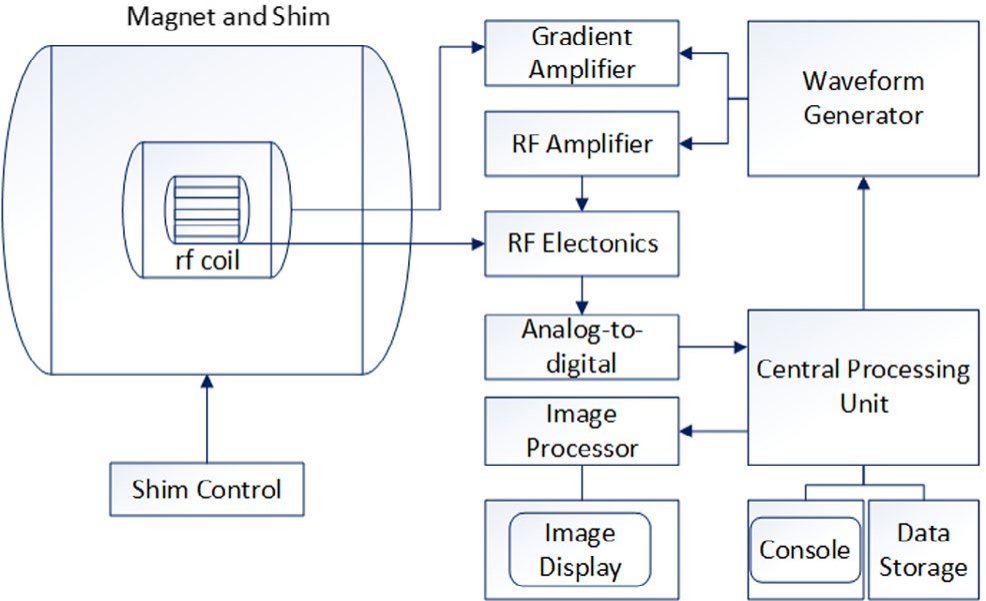
Schematic diagram of MRI

Magnetic resonance imaging (MRI) allows early detection of familial BC regardless of patients’ age, breast density or risk status.^39^ Figure 1 is schematic diagram of MRI. Water dispersion coefficient of different tissues exists with differences. Magnetic resonance diffusion weighted (MRDW) is a technique that can show clear movement of water molecules in the body. Therefore, MRDW has become a method for diagnosing BC patients. Through literature review, we found that malignant tumours have typical water diffusion‐limited effects in comparison with benign tumours, so researchers can distinguish benign and malignant breast tumours by using MRDW to measure apparent diffusion coefficient (ADC) values (represent diffusion‐limited effects) of tumours (ADC values: normal breast group > benign group > malignant group).^40^, ^41^ Recently, a review reported that the optimal threshold values for ADC in distinguishing benign and malignant lesions are as follows: 1.06 × 10^−3^ mm^2^/s ~ 1.10 × 10^−3^ mm^2^/s.^42^ Dynamic contrast‐enhanced MRI (DCE‐MRI) has higher resolution of soft tissues than MRDW, and it can clearly show morphological characteristics and haemodynamic characteristics of the lesions in vivo.^43^ Researchers found that the positive predictive value (98%) of DCE/MRI is higher than the positive predictive value (77%) of MRI alone, and the specificity points to 97%.^44^ Guindalini^45^ compared the diagnostic techniques of BC and found that biannual DCE‐MRI and annual MG for BC patients have performed well and low recall rates. Magnetic resonance spectroscopy (MRS) is a non‐invasive method that can also improve diagnostic rate of BC, by evaluating the risk of BC and guiding treatment of BC.^46^, ^47^

Magnetic resonance elastography (MRE) is another special magnetic resonance technology that can provide information on tissue elasticity by transmission of mechanical waves in tissues. Bohte et al^48^ elaborated MRE’s future tendency that it can delineate pre‐operative tumour and predict response to treatment and metastatic potential of primary tumours. PET/MRI, Positron emission computed tomography (PET) combined with MRI, can display soft tissue structures of the breast and chest wall. PET can provide molecular‐level information in vivo, and PET/MRI can improve the positive predictive rate of patients and has great value in evaluating BC metastasis.^9^, ^49^, ^50^

Magnetic resonance imaging is an auxiliary method that has many advantages in diagnosing BC. However, there are many factors that influence the wide application of MRI, such as long imaging time, high cost, cannot be carried out if has metal material in patient's body, and so on. Therefore, MRI can be used in situations where the primary BC is too small, where all information about tumour needs to be obtained, and for screening of high‐risk groups, etc In the future, MRI will tend to have higher signal‐to‐noise ratio, shorter imaging time and lower cost. Likewise, advancement in MRI should also consider how to reduce the use of contrast agents, so that it serves every stage of BC.

## 3 MOLECULAR BIOTECHNOLOGY EXAMINATION

Molecular biotechnology examinations can diagnose BC earlier than imaging techniques. Nevertheless, it cannot replace the imaging techniques and become auxiliary methods to diagnose BC. The purposes of molecular biotechnology examination are to analyse specific biomarkers such as nucleic acid, proteins, cells and tissues of patients. These examinations can help doctors obtain much clinical information at the molecular level. At present, these examination techniques mainly include nucleic acid hybridization system, real‐time fluorescence quantitative PCR system, protein hybridization system, flow cytometer, needle biopsy and immunohistochemistry (IHC). These techniques help us analyse BC from the level of nucleic acids, proteins and cells.

### 3.1 Novel specific biomarkers

Circulating tumour cells (CTCs) enter the blood circulation from primary tumour tissues, and the number of CTCs is about 1 ~ 10^2^/mL in peripheral blood. Jin et al^51^ investigated the viability of using CytoSorter^®^system to detect CTCs and to evaluate the diagnostic value of CTCs in BC. Their results showed that the CTCs can differentiate BC patients from the patients with benign breast diseases or healthy volunteers, as a diagnostic aid for early cancer diagnosis and cancer staging.^51^ CTCs could be used as a novel biomarker in assisting BC detection.

Circulating tumour DNA (ctDNA) is fragments of tumour genomic DNA that contains characteristics of gene variations consistent with primary solid tumour. ctDNA is thus very helpful in identifying the DNA from tumour cells or normal cells, as the number of ctDNA is very small in peripheral blood. Thus, the quantitative and qualitative detection methods for ctDNA are based mainly on PCR and next‐generation sequencing (NGS). Ma et al^52^ had a longitudinal monitoring of 21 patients during treatment that showed that the molecular tumour burden index (mTBI, a measure of the percentage of ctDNA in samples), positively correlated with tumour size as evaluated by computed tomography (*P* < .0001, Pearson *r* = .52), and detected disease progression 8‐16 weeks.^52^ Therefore, ctDNA could be used to assess tumour heterogeneity and predict treatment outcomes in metastatic BCs.^52^

Exosomes are membrane‐enclosed phospholipid extracellular vesicles with a variety of tumour antigens which can be applied in the diagnosis and treatment of cancer due to their high secretion on the surface of cancer cells.^53^ Exosomes have stable chemical properties, and their size is 30‐150 nm.^53^ Ni et al^54^ investigated whether the enrichment of miRNAs in exosomes reflects the pathogenesis of BC and ductal carcinoma in situ (DCIS). The levels of exosomal miR‐16 were higher in plasma of BC (*P* = .034) and DCIS (*P* = .047) patients than healthy women and were associated with oestrogen (*P* = .004) and progesterone (*P* = .008) receptor status. Moreover, lower levels of exosomal miR‐30b were associated with recurrence (*P* = .034), and exosomal miR‐93 was upregulated in DCIS patients (*P* = .001).^54^ Taken together, their result showed that different signatures of miR‐16, miR‐30b and miR‐93 in exosomes from BC and DCIS patients are associated with a particular biology of breast tumours.^54^ Therefore, exosomes have become a research hotspot in recent years because of their great diagnostic potential.

Long noncoding RNA (lncRNA) can involve in the regulation of cell cycle of tumour cells and a variety of cell signalling pathways of cancer cell invasion, metastasis, resistance of chemotherapy and so on. Shao et al^55^ found two lncRNAs that significantly correlated with outcomes of breast cancer and were regulated by methylation status. Liang et al^56^ revealed that RHPN1 antisense RNA 1 (RHPN1‐AS1) was induced by KDM5B and promoted BC via RHPN1‐AS1/miR‐6884‐5p/ANXA11 pathway. Besides, H19, an oestrogen‐inducible lncRNA, was reported to function in the cell survival and proliferation, which was from the oestrogen in breast cancer cells.^57^ Therefore, the functions of lncRNAs in initiation, progression and metastasis of breast cancer are emerging and are expected to be a potential new diagnostic marker and therapeutic target for BC.^56^

Circular RNAs (circRNAs) were recently discovered as a looped subset of competing endogenous RNAs, with an ability to regulate gene expression by microRNA sponging.^58^ Lu et al^59^ found that a total of 715 circRNAs were notably overexpressed, and 440 were remarkably downregulated in the BC lesions compared with healthy tissue samples among 1155 differentially expressed circRNAs. In 2019, Yan et al^60^ introduced hsa_circ_0072309 as a novel prognostic biomarker in BC, which is a miR‐492 sponge that is downregulated in BC. Dysregulation of this circRNA increases proliferation, migration and invasion in BC cells, and thus, it has a potential role in BC, as it is highly conserved and stable.

In all, these novel biomarkers not only are monitored dynamically, but are also used to judge prognosis. The patient's body fluids are used as samples for CA biopsy.

### 3.2 Nucleic acid hybridization system

#### 3.2.1 Nucleic acid hybridization

Nucleic acid hybridization techniques mainly include fluorescence in situ hybridization (FISH) and aptamer probe hybridization (APH). They can find special fragments of tumour biomarkers and search new tumour biomarkers when diagnosing BC.

**Figure 2:**
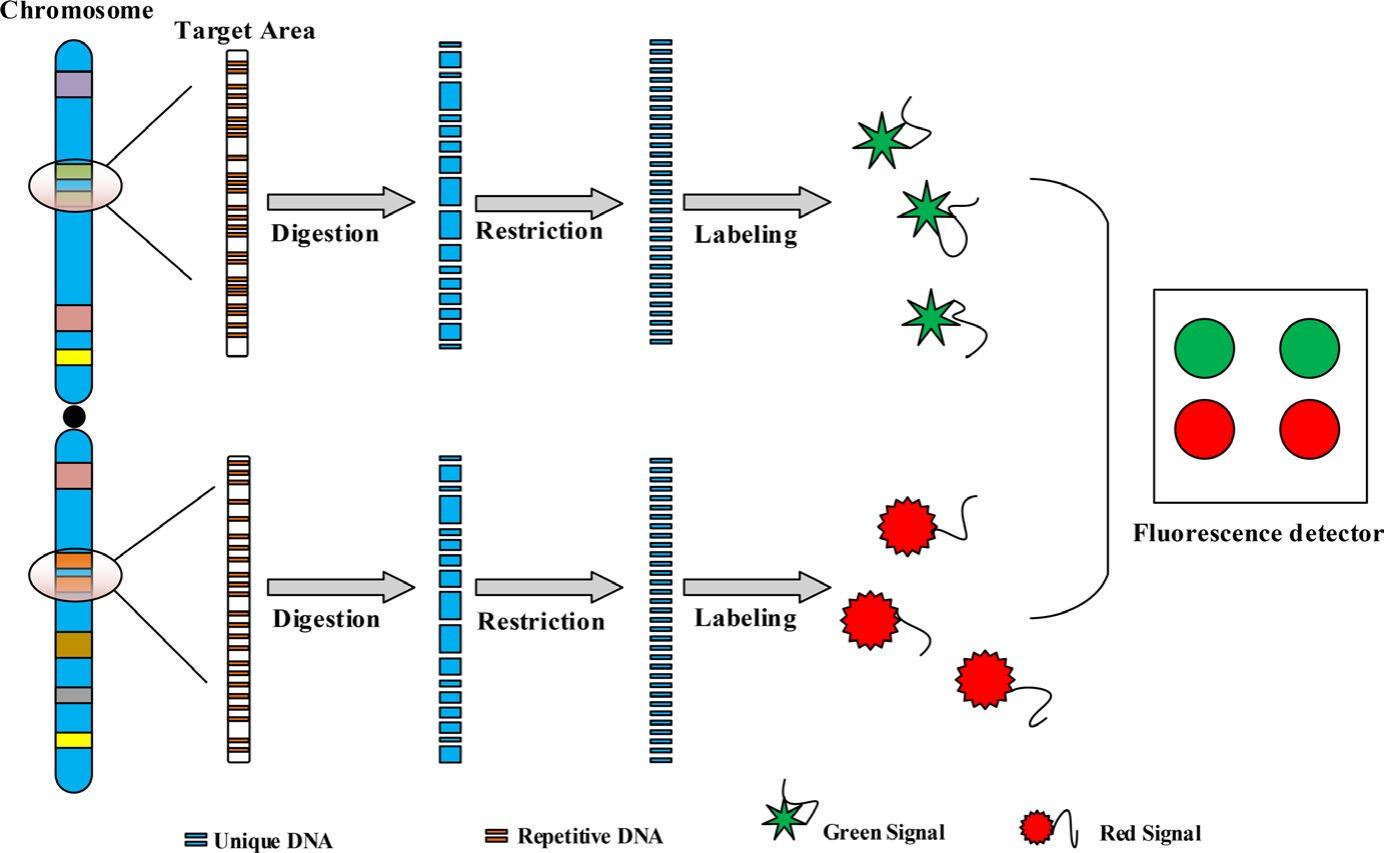
Technical principle of fluorescence in situ hybridization

FISH has made huge contributions to the development of molecular biology diagnostics.^61^ Its principle follows (Figure 2) base pairing. These data display that approximately 25‐30 per cent of all BC are human epidermal growth factor receptor 2 (*HER‐2*)–positive BC.^62^, ^63^ FISH has high response rates (2474 of 2524; 98.0%) to amplify *HER‐2* gene and has high *HER‐2* copies number per cell (by 2.86; *P* = .02).^64^ FISH detection is an important factor in whether a medication (Herceptin) is needed or not for BC patients. Meanwhile, FISH is considered the “gold standard” for detecting whether the *HER‐2* gene is activated.^65^ In addition, FISH shows other advantages, including reproducibility, stability and high sensitivity. However, these factors limit its promotion, including the need for complex probes design and special fluorescence detector. In the future, multicolour fluorescence in situ hybridization will be a tendency in greatly improving the throughput when searching genetic sites.

**Figure 3:**
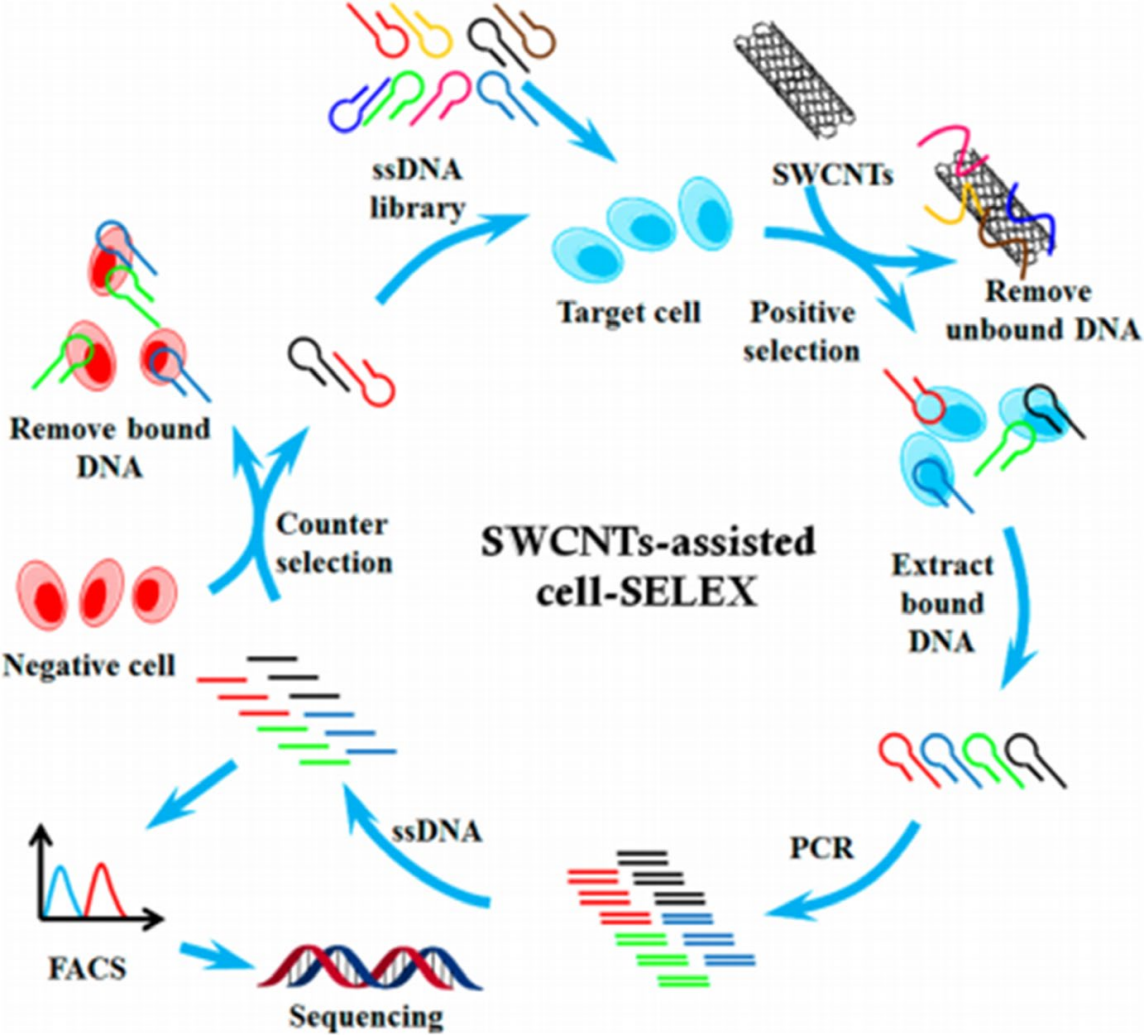
Schematics of cell‐based aptamer selection.^147^ (Reproduced with permission from Copyright 2014, American Chemical Society)

Aptamer probe hybridization is another highly sensitive and specific technique. Suitable aptamers are key factors in the accuracy of APH. These aptamers mainly are produced by Systematic Evolution of Ligands by Exponential enrichment (SELEX).^66^ At present, Cell‐SELEX is one of the most representative of SELEX, and it has become the main method that gets optimal aptamers from tumours.^67^ The schematic diagram of Cell‐SELEX is shown in Figure 3. Suitable aptamers can identify some specific fragments that can be used to diagnose diseases. Kim^68^ prepared a nucleotide aptamer (SE15‐8‐QDs) for detecting BC and found that it is more sensitive than the common probes. Cai^69^ developed a new type of fluorescence aptamer (AAI2‐5) that can detect MCF‐7 BC cells and MDA‐MB‐231 cell lines easily and sensitively from breast cells with an accuracy of 90%. However, the process for obtaining suitable aptamers or probes is complex and difficult, requiring a lot of time and money, and is not suitable for promoting to use in primary hospitals. In the future, APH will have the easy process for screening suitable aptamers and will find more biomarkers of BC.

#### 3.2.2 Gene chip and next‐generation sequencing

**Figure 4:**
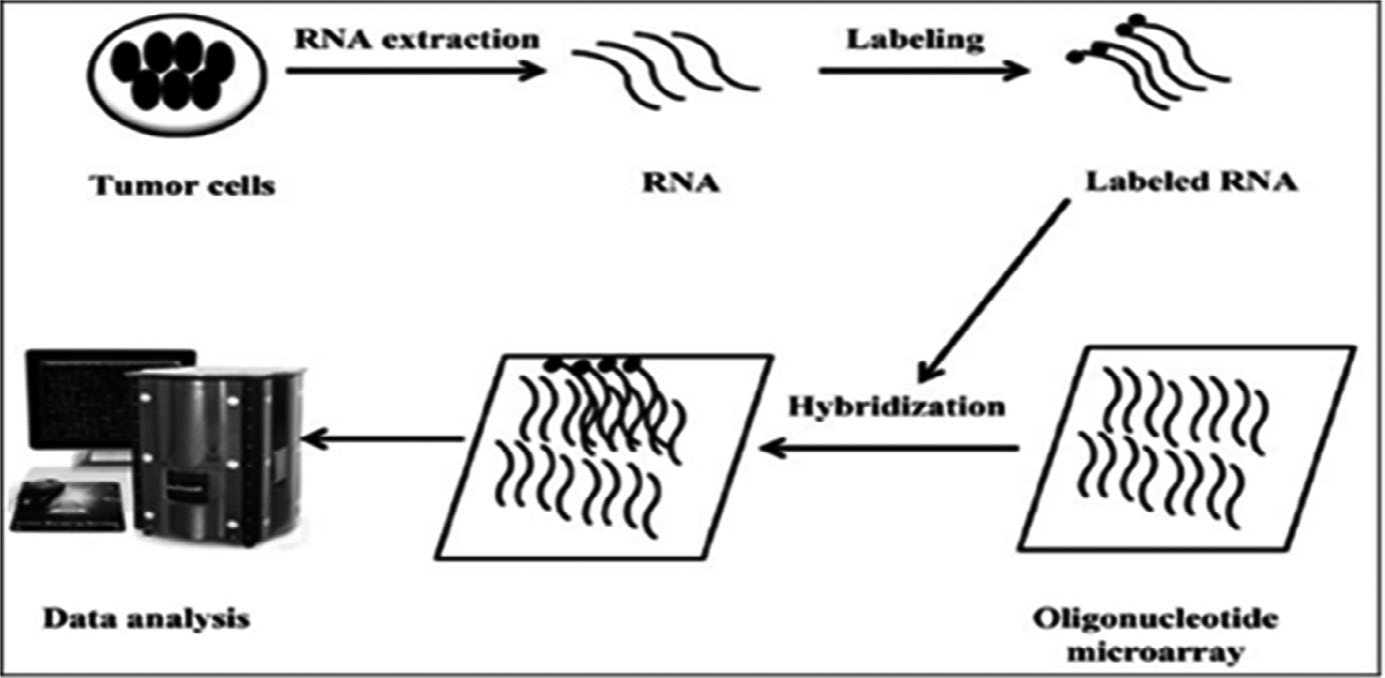
Flow chart of microarray technology.^162^ (Reproduced with permission from Copyright 2012, Rajnish Kumar)

Gene chip can analyse a large number of fragments of nucleic acid simultaneously, and it is applied widely in diagnosing BC. Gene chip is used to observe and analyse the condition of nucleic acids in BC cells or tissues and also can find new diagnostic biomarkers for BC by screening a large number of samples. As well known, gene chip is essentially a high‐density oligonucleotide microarray.^70^, ^71^, ^72^ At present, there are two methods for chip preparation: in situ synthesis and direct point method.^73^ However, in situ synthesis is the main method, and its schematic diagram is shown in Figure 4. Using gene chip technology, researchers found mechanisms for doxorubicin resistance in BC and screened these key genes for BC therapy.^74^ Jiang et al^75^ used LIMMA (Linear Models for Microarray Data) methodology to identify differential expression of lncRNAs between tumour and normal samples, and they identified 26 inter‐genic lncRNAs transcripts that were specifically expressed in tumour cells [*P* < .005, FDR < 0.15]. There are however limitations of gene chip, such as difficulty in synthesizing probes, easy appearance of positive signals and especially complicated nucleic acid extraction. In the future, with development of nanotechnology, the size of chip will be smaller and the throughput of gene chip will be higher.

**Figure 5:**
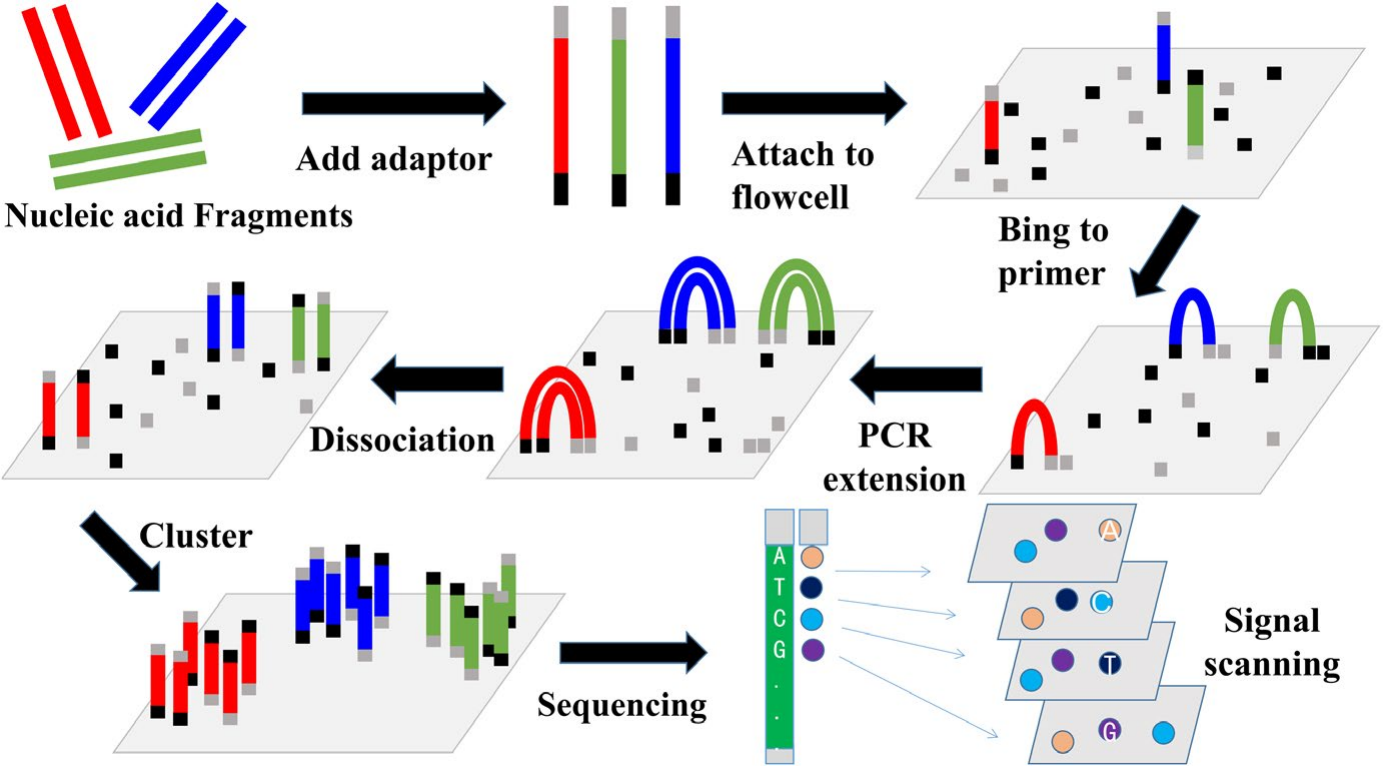
Schematic diagram for NGS

Next‐generation sequencing (NGS) was put forward by Metzker.^76^ The schematic diagram of NGS is shown in Figure 5. This technique makes great contribution to get the genome sequence information and can help find mutant gene sites. Currently, NGS has been applied widely in diagnosing BC. For example, Dong et al^77^ designed targeted NGS platform and found three additional possible disease‐causing mutant genes. Liang et al^78^ found twelve common mutant genes by NGS detection, namely *TP53, PIK3CA, MYH9, NOTCH2, BRCA2, ERBB4, FGFR3, POLE, LAMA2, ARID1A, NOTCH4* and *ROS1*, in inflammatory BC. Moreover, Kim et al^79^ detected at least one somatic mutation in 44 of 61 tDNA (72.1%) and 29 of 44 (65.9%) and cfDNA, and the overall concordance rate of cfDNA to tDNA was 85.9%, utilizing next‐generation digital sequencing technology. Wu et al^80^ used RNA sequencing to detect tumour‑specific miRNAs, and their results showed that the exosome levels of hsa‐miR‐150‐5p, hsa‐miR‐576‐3p and hsa‐miR‐4665‐5p were higher in BC with recurrence compared to those in patients without recurrence. Page et al^81^ used a novel targeted NGS panel to examine cfDNA to detect somatic mutations and gene amplification in women with metastatic BC. Their results showed no mutations were identified in cfDNA of healthy controls, whereas exactly half the patients with metastatic BC had at least one mutation or amplification in cfDNA (mean 2, range 1‐6) across a total of 13 genes.^81^ Scarpitta et al^82^ screened the 24 genes involved in BC predisposition, genome stability maintenance and DNA repair mechanisms by NGS and found that a positive family history is a strong predictor of germline BRCA2 mutations in male BC. Ou‐Yang et al^83^ compared differences in gene expressions in parental and CHD4‐deficient cells by NGS and suggested that the chromodomain‐helicase‐DNA‐binding protein 4 regulates β1 integrin in triple‐negative BC. However, the main limitation of NGS is short reads of about 200‐500 bp. Single‐molecule sequencing can offer long read lengths, direct RNA sequencing, direct identification of base modifications and so on, but at present NGS can easily occupy mismatch and is not suitable for analysis of satellite DNA.^84^ Therefore, sequencing can help us to analyse the gene mutations in humans and can predict the risk of BC. Research shows that NGS will be main trend of high throughput, high accuracy and fewer mismatch in the future.

### 3.3 Real‐time fluorescence quantitative PCR system

Real‐time fluorescence quantitative PCR (RT‐qPCR) system can monitor the process of nucleic acids amplification and predict the protein expression condition. Various biomarkers, such as cfDNA, ctDNA, lncRNA, circRNA, microRNA and so on, have been expressed in BC, but their content is too low to be detected by ordinary instruments. Therefore, RT‐qPCR system is a good choice and can predict risk of BC by analysing the level of mRNA expression. It has some advantages, such as less time consumption, high sensitivity and specificity. In addition, it requires less samples and shorter analysis time compared with other molecular methods.^85^ Meanwhile, RT‐qPCR is optimal technology for identifying difference of expression levels of mRNA between malignant tumours and normal tissues.^86^ Mansoori et al^87^ found that *Bach‐1* mRNA was overexpressed, while miR‐142‐3p was downregulated in the BC tumours and then summed up that the expression of miR‐142‐3p has relationship with BC. Moreover, RT‐qPCR system can also guide BC treatment by monitoring specific expression of mRNA.^88^, ^89^ Matouk et al^90^ used the system to analyse the expression condition of *H19* gene in BC patients and healthy individual and found the expression difference between them, indicating that the *H19* gene is a potential molecular marker for diagnosing BC. However, to obtain satisfactory results, high‐quality mRNA should be extracted. The process for extraction of high‐quality mRNA is difficult because of presence of RNase in the environment.^91^ So the full‐automatic nucleic acid extraction device appears and will improve the RNA yield for getting the accurate analysis results.^92^

**Figure 6:**
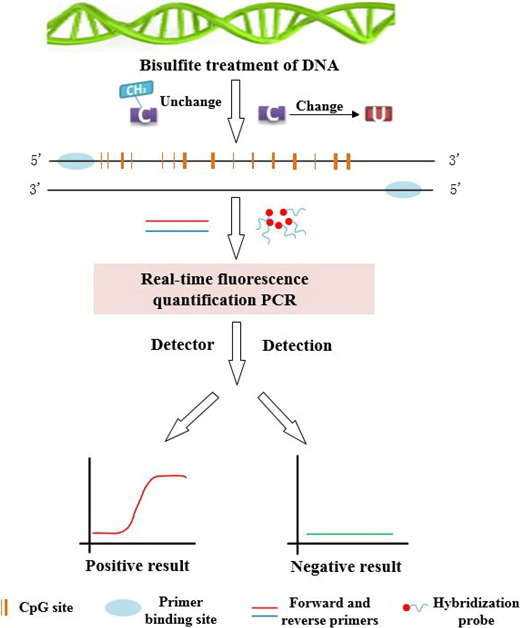
Schematic diagram of MethyLight

Gene promoter region DNA methylation can also cause cancer, because it can produce similar effects to gene mutations, such as obtaining or losing functions of some specific genes.^93^, ^94^ Methylation‐based RT‐qPCR system is widely used for analysing genetic methylation. The Table 2 lists part of methylation genes in BC. In order to understand the detection process for methylated genes, we elaborate it by Figure 6. Next, the applications of methylation in BC are expounded. Luo et al^94^ identified that these genes, *ALDH1L1*, *HOPX*, *WNT5A* and *SOX9*, were hypomethylated after neoadjuvant chemotherapy (NAC) treatment by using MethyLight ddPCR and the methylation levels of 4 genes in BC patients after NAC were lower than those before NAC. MethyLight can be used to research expression conditions of methylated silencing genes in cell lines during treatment of BC with drugs.^95^ Mastoraki et al^96^ considered that methylation of *ESR1* gene can become a potential liquid biopsy‐based biomarker to evaluate the risk of BC and *ESR1* methylation in CTCs and was associated with response of everolimus/exemestane. In addition, the MethyLight can explore chemo‐resistance to breast tumour by analysing methylation gene.^97^ Therefore, MethyLight plays very important part in diagnosing BC. However, MethyLight has some limitations; for example, the nucleic acid needs to be treated (totally methylated or un‐methylated nucleic acid), needs to design complex probes and requires professional operation. In the future, integrating extraction and methylation detection of DNA will be a tendency, which will not only improve the DNA yield, but also the efficiency of methylation.

**Table 2:** Partially methylation gene in breast cancer

Gene	Gene description	References
*BRCA1*	*BRCA1* gene is a tumour suppressor, and it can maintain genomic stability. The nuclear phosphoprotein is encoded by *BRCA1* gene. Methylation of the *BRCA1* gene promoter region can change expression of *BRCA1* gene and loss function of tumour suppressor	^148^
*E2F4*	*E2F4* gene is potential basal transcription factor, and it can promote tumour growth. Methylation of *E2F4* gene can cause upregulation expression of *E2F4* gene and accelerate the development of tumours	^149^
*PITX2*	*PITX2* gene is a prognostic marker for progesterone receptor‐positive patients, and it is closely associated with poor survival and distant metastasis of breast tumours. If *PITX 2* gene is methylated, it can be considered low risk of distant metastasis recurrences and need not adjuvant chemotherapy	^150^
*Hox*	The methylation of *Hox* gene is closely related to the high expression of oestrogen and progesterone receptors, and methylation of *HoxD13* gene is closely related to breast tumour size and poor clinical treatment	^151^
*AKT1*	Methylation of *AKT1* gene is observed to be associated with BC, and it affects expression of *AKT1* gene. The expression of *AKT1* gene has significantly associated with HER‐2 protein status	^152^
*Soxl7*	*Soxl7* gene has significantly associated with breast tumour size and lymphatic metastasis, but un‐methylation of *Soxl7* gene is found in normal breast tissue and serum	^153^
*CDKN2A*	The methylation of *CDKN2A* gene in patients with malignant tumour is found, but un‐methylation of *CDKN2A* gene is found in patients with benign breast disease. Methylation of *CDKN2A* gene also is associated with distant metastasis of breast tumours	^154^
*FHIT*	*FHIT* gene is widely expressed in normal tissues, and methylation of *FHIT* gene occurs in 31% of patients with primary BC. In particular, after *FHIT* gene is methylated, its expression quantity is changed in patients with sporadic ductal carcinoma	^155^
*TIMP‐3*	Methylation of *TIMP‐3* gene is found in BC cells, but does not find in normal tissues. The degree of methylation of *TIMP‐3* gene is positively correlated with malignancy of BC	^156^
*MDGI*	The *MDGI* gene is also lowly expression in BC tissues. If promoter region of *MDGI* gene is methylated in breast cancer patients, methylation of *MDGI* will be only slightly influenced by surgery, whereas tamoxifen therapy will be a more pronounced effect	^157^
*RASSF1A*	In the patients with sporadic BC, finding 33.3% of *RASSF1A* gene was deleted or methylated	^158^
*HSD17B4*	Methylation of *HSD17B4* gene is an independent predictive marker for pathological complete response in some studies. If the *HSD17B4* is not methylated in patients with BC, these patients will be not benefit from trastuzumab treatment, but will be benefit from lapatinib treatment	^159^
*ESR1*	Abnormal hyper‐methylation of *ESR1* gene is found in BC cells, and it will hope to become a new biomarker of breast tumour	^160^
*RhoBTB2*	Aberrant methylation of *RhoBTB2* gene may affect expression of the *RhoBTB2* gene, which influences PR protein status, become the factor that induce BC	^161^
*NBPF1*	Hypermethylation of promoter region of *NBPF1* gene is found in patient's serum or plasma with BC, and thus, the *NBPF1* methylated from patient's serum or plasma may become potential tumour biomarker for detection of BC	^160^

### 3.4 Protein hybridization system

Tumour cells or tissues contain not only the nucleic acids but also many proteins. The “central dogma” of molecular biology shows that proteins are closely associated with nucleic acids. However, if the final protein has no change, the differential expression of nucleic acids may not cause cancer. Therefore, proteins are another important biomarker for diagnosing cancers and analysing the situation of proteins can predict occurrence of cancer. Similarly, proteins, as important biomarker, make great contribution to diagnosis of BC. In Table 3, we introduce the most common and latest oncogene proteins involved in BC. These proteins can be quantitatively evaluated by immunochemistry, RT‐qPCR and Western blot. The difference between them is the different detective object, in which RT‐qRCR is for mRNA and Western blot and immunostaining are for proteins.

**Table 3:** Partial oncogene proteins related to breast cancer

Protein	Protein description	References
HER‐2	HER‐2 as therapeutic and prognostic biomarker plays a significant role in Human BC. It is found that adenomas and carcinomas have higher levels of HER‐2 protein than normal mammary glands	^163^
CA125	CA125 as a predictive marker of ovarian/breast carcinoma, it depends on disease nature/stages. CA125 plays an interactive role in the disease processes, and it is closely related to BC	^164^
CA19‐9	Levels of CA19‐9 are correlated with treatment response and survival of BC	^165^
MUC1	MUC1‐MBP is a member of the mucins family, and it is present in normal glandular epithelial cells and tumour cells. MUC1‐MBP consists of a polypeptide core and a side chain sugar chain. MUC1‐MBP widely distributed on the surface of BC cells	^166^
ER	ER in the pathophysiology of BC plays an important role, and it as an index can be used to guide pharmacy for BC patients	^167^
CypB	BC tissues have higher levels of CypB proteins than para cancerous tissues. Functional study confirms that downregulation levels of CypB may inhibit tumour cell growth, proliferation and migration	^168^
CA153	When the breast becomes cancerous, the activities of protease and salivary enzyme are increased, causing destruction of the cytoskeleton of the gland, causing CA153 saccharide antigen generally separated from the cancer cell membrane and releasing into the blood. It is an important index for screening BC	^169^
CEA	CEA is an acidic glycoprotein with a specific determinant of human embryonic antigen. It is a broad‐spectrum tumour marker that can be expressed in a variety of tumours. It is also elevated in the serum of patients with BC, lung cancer and other malignant tumours	^170^
PR	Analysis of PR proteins remains controversial in BC. The level of PR + is related to age of BC patients. The deletion of PR proteins might cause BC	^98^

#### 3.4.1 Immunochemistry

**Figure 7:**
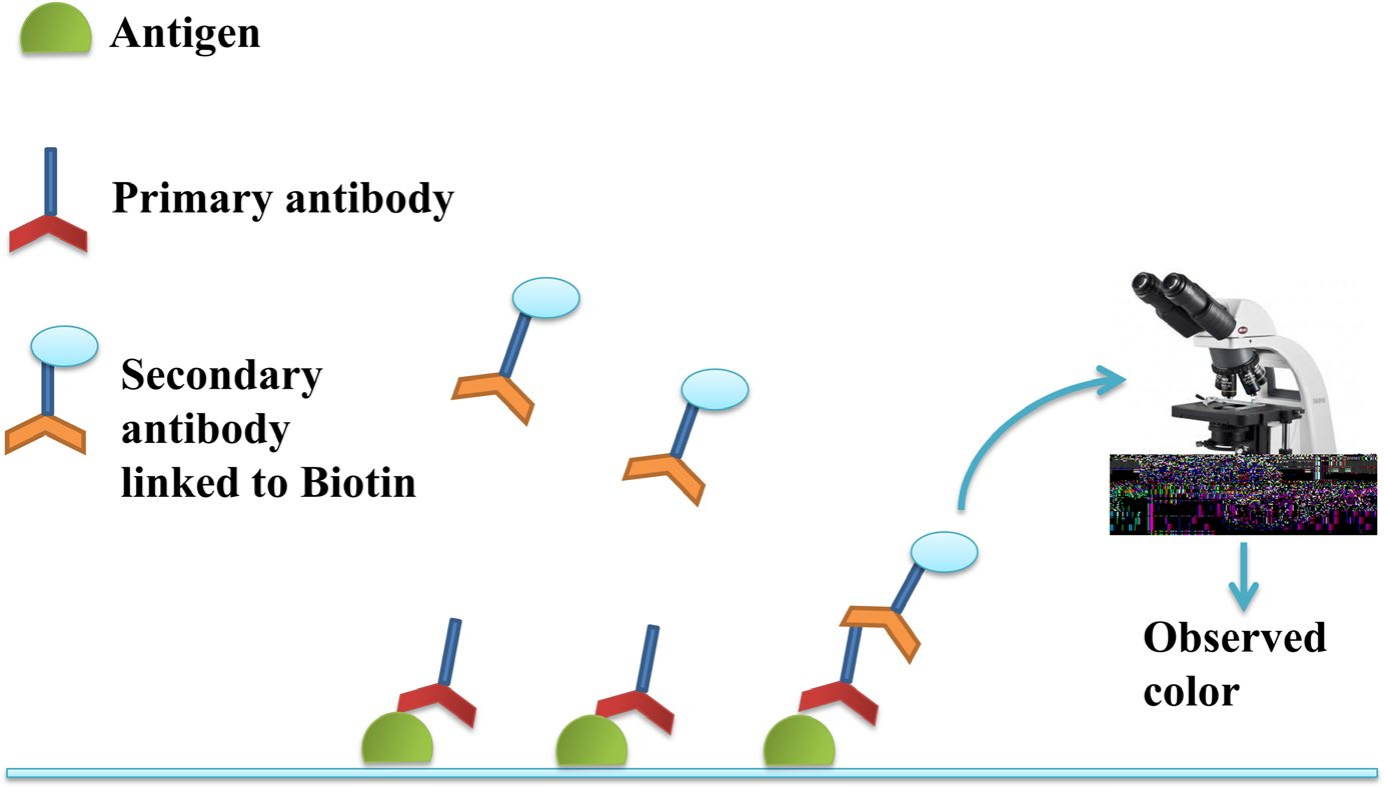
Schematic diagram of immunohistochemical principle

For pathologists, immunostaining (IHC) can accurately locate the site of organization and is an auxiliary method for diagnosing BC. IHC analysis of breast tumours has advantages in the following four aspects: (a) can distinguish between benign and malignant breast tumours; (b) can assess interstitial infiltration; (c) can distinguish between ductal and lobular tumours; and (d) can detect expression of proteins associated with BC treatment and prognosis, to guide endocrine therapy and prognosis.^98^, ^99^, ^100^, ^101^ At present, IHC is the best diagnostic method for oestrogen receptor (ER) and progesterone receptor (PR) in BC.^102^ The basic principle of IHC (as shown in Figure 7) is antigen‐specific binding of antibodies, and these antibodies are usually labelled with colour reagents (such as fluorescein and metal ion) to detect the antigen, protein, peptides, etc IHC can screen and diagnose BC patients by evaluating the level of marker proteins.^103^ *HER‐2* gene amplification may cause overexpression of *HER‐2*, so Suryavanshi et al^104^ used IHC to confirm whether *HER‐2* gene was amplified abnormally in BC patients by detecting the level of protein and evaluating that the effect of IHC was close to FISH. Surely, IHC also can help researchers to explore the relationship between external factors and BC. For example, Wang et al^105^ found the underlying association between alcohol and BC by utilizing IHC. Toomey^106^ used IHC to assess the level of *PTEN* protein and found that 14 of 45 (31.1%) tumours samples had low (absent or weak) *PTEN* expression and PR‐negative tumour had higher *PTEN* expression than PR‐positive tumours (37.9% vs 18.8%) in BC. BC formation may cause changes of protein levels, so IHC is able to study the mechanism of breast tumours by analysis of protein levels. However, IHC needs fluorescence labelling which is time‐consuming and difficult to prepare.

#### 3.4.2 Western blot

Similarly, Western blotting also utilizes the antigen‐antibody binding character that is highly specific. On one hand, the capacity of histological localization of Western blotting is poorer than that of IHC, but the capacity of quantitative protein level is more accurate than that of IHC. On the other hand, for RT‐qPCR, though they all evaluate quantitatively the level of proteins, their detection objects are different, where RT‐qPCR is for nucleic acids, and Western blotting is for proteins. Using exogenous proteins to study proteins’ interaction is a common approach, but the most rigorous approach is to detect interactions between endogenous proteins.^107^ Western blotting can satisfy this need and be used in diagnosing BC. For example, Zhou et al^108^ used Western blot to investigate the expression of *UCA1* and microRNA (miRNA) in BC cells in response to *IMP1* expression. Liu used this technique to analyse the relationship between miRNA and *IDH1*gene. These results showed that Western blot can not only explore whether the proteins are expressed, but also verify whether the protein expression is abnormal.^109^ De Francesco et al^110^ found by Western blotting analysis that *HIF‐1α* and *GPER* expressions increased with time in CAFs cells, but expression decreased over time in SKBR3 cells. Moreover, Ansari^111^ utilized Western blot to analyse the level of proteins in BC and found that 191 from 1110 (17%) in the discovery set and 268 from 1554 (17%) in validation sets of cases had positive SLC7A5 expression (>15 H‐score), while 1019 in 1923 (53%) from metastatic BC cases had high mRNA expression (log_2_ intensity > 8). Surely, Western blot has deficiencies, such as use of expensive agents, easily false positive and needs professional operation. In the future, the decrease in price of Western blot agents will be a tendency and simply the process of Western blot operation.

### 3.5 Flow cytometer

**Figure 8:**
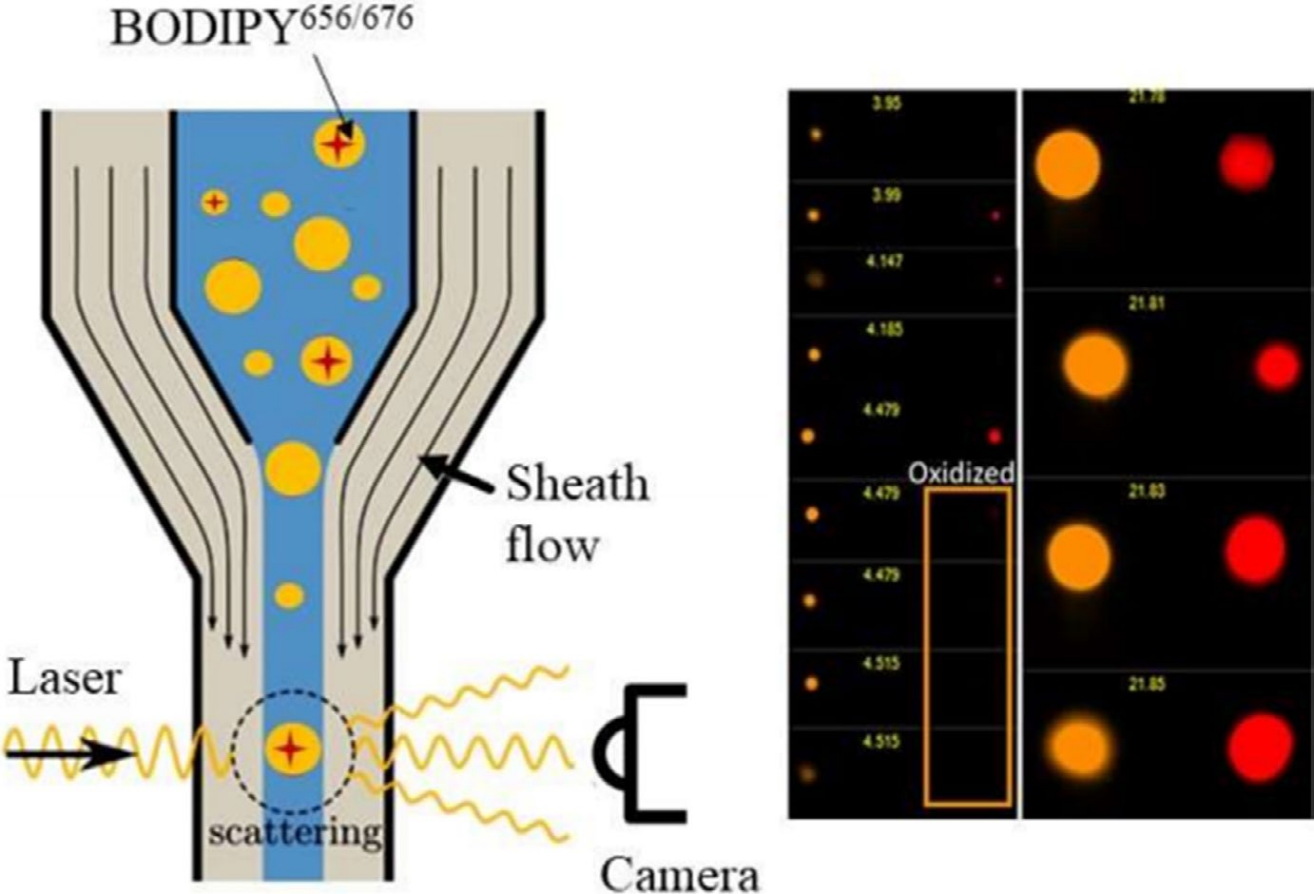
Isolation of individual droplets by flow cytomtry.^121^ (Reproduced with permission from Copyright 2020, American Chemical Society)

Flow cytometer (FCM) can reflect multiple physical characteristics of a single cell when the cell flows in suspension,^112^ and it has become an indispensable technology in diagnosis of BC. FCM is a high‐tech developed in the 1960s, and it is the combination of many disciplines and technologies, such as cytochemistry, immunology, materials science, molecular biology, spectroscopy, optical system, fluidic system, laser technology and computer technology.^113^, ^114^, ^115^, ^116^ Surely, FCM also has sorting function for tumour cells and can rapidly detect cells or biological particles through the one‐by‐one flow state, multi‐parameters or rapidly qualitative and quantitative analysis.^117^, ^118^, ^119^, ^120^ Figure 8 shows how marked sites on cell surface are detected by FCM.^121^ In the FCM, the cells or biological particles need to firstly be treated and labelled, so that they can be detected by laser.

In recent years, FCM, by combining with other detecting techniques, can achieve quantitative detection of low‐abundance genes.^122^ FCM also is excellent method in diagnosing BC and guiding medication. Kim et al^123^ used FCM to analyse tumour cell surface markers and found that hypoxic tumour microenvironment may associate with promoting malignant progression and therapy resistance. Chamberlin^124^ utilized the FCM to mark different cells and found that the ratio of luminal and basal cells presents a significant increase in obese mammary glands with weight gain and expounded how obesity is linked to BC. Moreover, Tu et al^125^ used FCM to trace these tumour cells and found that primary breast tumour growth cannot be affected by an oral administration of an FUT inhibitor (2‐fluorinated‐peracetyl‐fucose), but these medicines greatly reduce the lung metastatic. Through FCM analysis, Xu et al^126^ identified the origin of cancer stem cell (CSC)–like cells that would be critical to cancer treatment and found these breast non‐stem cancer cells are transferred to breast CSC‐like cells in apoptosis process. FCM not only can detect the biomarkers of BC cell, but also can also detect BC cells based on morphology. Patel et al^127^ utilized digital holographic cytometry (DHC) and found that a special marker, sialic acid‐molecularly imprinted polymers (SA‐MIPs), has impact on different BC cells’ morphology and motility. Similarly, Farghadani et al^128^ investigated the mechanism of inhibitory and cytotoxic activity of anticancer agent on BC cells, cell cycle progression using flow cytometry analysis, and found some valuable medicine.

There are some advantages of FCM, including nonspecific binding in antigen antibody may cause the signalling pathway of FCM to be affected. Dyestuff pollution in FCM experiment is also a big trouble, and expensive instruments are required. In the future, it is most important that diagnostic scheme for FCM should be standardized and agents of high efficiency and low cost should be developed.

### 3.6 Puncture biopsy system

Needle biopsy is a main method to obtain tumours tissue or cells sample for histopathological diagnosis. These puncture biopsies system include fine‐needle aspiration cytology (FNAC), core needle biopsy (CNB) and vacuum‐assisted breast biopsy (VABB).^129^, ^130^ At present, VABB has excellent effects in the auxiliary diagnosis of BC. Its advantages are as follows: single puncture can accurately and simply collect many samples, accurate positioning, convenient operation, smaller trauma area and so on. In general, obtaining samples (cells or tissues) by puncture needs staining (usually using haematoxylin‐eosin) to easily observe samples under optical microscope, which can rapidly analyse and identify pathological tissue and cell morphology to help doctors make pathological diagnosis. Surely, these samples also are detected by other molecular biology methods. Zhang et al^131^ used high‐frequency ultrasound‐guided breast mass biopsy to diagnose BC in two hundred patients. Their results showed that each patient had a successful puncture rate of 100% under the guidance of ultrasound. Moreover, no complications occurred, and 95% (190/200) of the patients were clearly diagnosed, and 5% (10/200) were orientally diagnosed. The biopsy examination results were completely consistent with surgical pathological results in 170 patients, accounting for 85%. Thus, this method can provide strong evidence for diagnosis and identification of benign and malignant breast tumours, and for choosing the correct operation scheme.^131^ Hu et al^132^ performed US‐guided fine‐needle aspiration biopsy (FNAB) for early‐stage BC and found that its sensitivity and specificity were higher than for US alone, 11.9% and 21.7%, respectively. With development of imaging, the accuracy of puncture biopsy is higher under imaging guidance. Guo et al^133^ offered a new integrated precise re‐biopsy algorithm for pathological confirmation and surveillance of recurrent BC. The technology is more sensitive and accurate than conventional imaging technologies in diagnosis of early‐stage BC.

However, there are some disadvantages of needle breast biopsy; for example, it may cause tumours transfer and researchers thought that high‐grade, non‐coaxial biopsies, triple‐negative BCs and multiple insertions may be risk factors for neoplastic seeding.^134^, ^135^, ^136^, ^137^ In the future, with development of biopsy needle, the risk of neoplastic seeding will be reduced and the accuracy of diagnosis will be improved. Surely, the latest imaging guidance will also promote the development of puncture biopsy.

## 4 CONCLUSIONS AND FUTURE PERSPECTIVES

In this review, we mainly introduced the common methods for diagnosis of BC. As the exploration of imaging technology goes deeper, researchers realize that the single imaging technology has lower accuracy and cannot meet the need for BC diagnosis, and the combination of various imaging modalities will be one of the major developing directions.^138^, ^139^, ^140^ Moreover, with development of biosensors, a lot of BC biomarkers have been found. The combination of imaging sensors and biosensors can get unexpected results.^141^, ^142^ Meanwhile, more and more aptamers are developed, which increases connection between imaging and molecular biology.^143^, ^144^ These aptamer‐functionalized nano‐composites not only can become indicators for imaging, but also can identify cancer cells, and/or even classify BC cells subsets. In another aspect, screening for new tumour biomarkers is still an important task which can help doctors diagnose BC faster and more accurately. Currently, proteins, nucleic acids and lipids are the main tumour markers in breast cancer, while the question remains whether single markers could not acquire definite diagnosis results.^145^, ^146^ Hence, multiple tumour markers or screening for a super new marker can greatly improve the positive diagnostic rate for BC and reduce the negative diagnosis rate.

Over the next few years, imaging instruments still will be the routine method for screening BC, because they suit to be widely applied. However, new markers for BC will advance these technologies to higher throughput, faster, higher sensitivity and specificity. In the future, with development and use of these techniques, they not only can diagnose BC from various aspects, but also can evaluate effect of treating BC. Of course, different types of BC also will be evaluated by corresponding diagnostic methods, to get the most accurate results.

## CONFLICT OF INTEREST

The authors have no conflict of interest.

## AUTHOR CONTRIBUTIONS

The article was written by Ziyu He and Zhu Chen. Miduo Tan, Sauli Elingarami, Yuan Liu, Nongyue He, Taotao Li and Wen Li contributed to conceptualization and revision of the article. The article was funded by Zhu Chen, Yan Deng, Song Li and Juan Fu. All authors reviewed the final manuscript.

## Data Availability

Some or all data, models or code generated or used during the study are available from the corresponding author by request.
